# Characteristics and Clinical Managements of Chronic Skin Ulcers Based on Traditional Chinese Medicine

**DOI:** 10.1155/2012/930192

**Published:** 2012-05-14

**Authors:** Fu-Lun Li, Yi-Fei Wang, Xin Li, Feng Li, Rong Xu, Jie Chen, Lin Geng, Bin Li

**Affiliations:** Department of Dermatology, Yueyang Hospital of Integrated Traditional Chinese and Western Medicine, Shanghai University of Traditional Chinese Medicine, Shanghai 200437, China

## Abstract

Chronic skin ulcer (CSU), including diabetic ulcers, venous ulcers, radiation ulcers, and pressure ulcers, remains a great challenge in the clinic. CSU seriously affects the quality of life of patients and requires long-term dedicated care, causing immense socioeconomic costs. CSU can cause the loss of the integrity of large portions of the skin, even leading to morbidity and mortality. Chinese doctors have used traditional Chinese medicine (TCM) for the treatment of CSU for many years and have accumulated much experience in clinical practice by combining systemic regulation and tropical treatment of CSU. Here, we discuss the classification and pathogenic process of CSU and strategies of TCM for the intervention of CSU, according to the theories of TCM. Particularly, we describe the potential intervenient strategies of the “qing-hua-bu” protocol with dynamic and combinational TCM therapies for different syndromes of CSU.

## 1. Introduction

A chronic skin ulcer (CSU) is defined as a wound lesion that lasts more than four weeks without remarkable healing tendency or as a frequently recurrent wound [1]. Traditional Chinese medicine (TCM) considers that CSU belongs to the “ulcer” branch of the Ulcer and Sore diseases. There are more than 8 million patients who have been diagnosed with CSU each year in the United States [2], which costs more than 10 billion dollars to treat this serious disease each year [3]. In China, patients with CSU account for 1.5%–3% of the total hospitalized patients in the surgical departments [4]. Therefore, the development of therapeutic strategies for the intervention of CSU patients is of great significance.

TCM has been used for the prevention and treatment of CSU for many years. Historically, there are several TCM theories for the intervention of CSU and they include the “wei-nong-zhang-rou (keeping the right amount of pus on the surface of the ulcer to stimulate the growth of granulation),” “qu-fu-sheng-xin (removing necrotic tissues to stimulate the growth of new skin),” and “ji-ping-pi-zhang (inhibition of inflammation to promote skin wound recovery).” These TCM theories have been used as the guidelines for the intervention of CSU. The principles of TCM intervention for CSU mainly focus on (1) systemic consideration, (2) treatment based on syndrome differentiation, (3) differentiation of diseases and determination of the disease stage, (4) combination of systemic with topical treatments, (5) interior and exterior treatments together, and (6) treatment of symptoms as well as the causes. Accordingly, a therapeutic method should not only facilitate the wound healing but also effectively decrease or relieve the scaring. Indeed, TCM has been used for the successful treatment of many cases with CSU. Here, we discuss the current approaches on TCM treatment of CSU, particularly by centering on the interventional strategies of “qing-hua-bu,” a dynamic and combinational therapy of TCM for different types of CSU.

## 2. Theoretical Understanding of CSU Development

In TCM, the pathogenesis of CSU is theoretically caused by “Re (heat) evil.” The pathogenic process of CSU was described first in “Lingshu: yongju” as follows: “cold evil accumulates in the meridian and results in a stiffness in blood flow and body jam, which inhibits the circulation of defensive energy, leading to inflammation. Subsequently, cold evil changes into heat evil, which causes tissue damages and then pus formation.” Accordingly, the damaged tissues in ulcers are the main factor contributing to the pathogenic progression of CSU. Conceivably, “removing necrotic tissues to stimulate the growth of new skin” has been used as a gold standard for the intervention of CSU in TCM [4]. This is consistent with the traditional view that “*the fresh skin will not grow until the removal of the necrotic tissues.*” Actually, “removing necrotic tissues to stimulate the growth of new skin” has been demonstrated to be successful in the treatment of CSU for many years in the clinic [5].

The “Fu (rotten)” tissues in the “Qu-Fu-Sheng-Ji” (removing necrotic tissues to stimulate the growth of new skin) protocol are comprised of infected necrotic tissues, overhyperplasia of pathological granulation and pathogens, which effectively obstruct the repair of an ulcer [5]. However, during the clinical practice, we found that treatment with TCM, according to the principle of “removing necrotic tissues to stimulate the growth of new skin,” had variable outcomes in patients with CSU, although they had ulcers with similar shape, color, secreted fluid, and location. Similar treatment resulted in the rapid recovery of ulcers in some patients, but did not in other patients. Although we always removed the damaged and necrotic tissues as much as possible, we did not observe the regeneration of fresh tissues, even with the recurrence of tissue damage in some patients. In the TCM history, there are many TCM doctors, who have investigated this phenomenon and described the potential reasons. In the Song dynasty, Ziming Chen described in his famous book of “wai-ke-jing-yao (essence of diagnosis and treatment of external diseases)” that “ulcer that has been treated for a long time without remarkable recovering displays the traits of white meat with little pus in the wound, which indicates a body deficiency in both Qi (vital energy) and Blood, a cold wound without sufficient qi and blood circulation.” Similarly, Dongxuan Buddhist in the Song dynasty wrote in the “wei-ji-bao-shu” (the guidelines of human health) that “if the carbuncle has progressed into ulceration, there are always “Yu” (obstructed blood supply and necrotic tissues) around the wound, which can invade into deep tissues to destroy muscles and bones. Hence, clearance of “Yu” should be of the primary strategy for the treatment of the disease.” Professor Hanjun Tang, a well-known Chinese doctor at the Department of Surgery of Longhua Hospital at Shanghai University of TCM, considers that the skin ulcer is a “Re (heat)” syndrome because the skin ulcers usually begin with “redness and swelling around the wound, which progressively forms pus.” In contrast, CSU is always at the middle or later stage of the disease process with the characteristics of “(1) the dim dark skin around the wound, (2) subsiden wound, (3) a little pus and secretion, and (4) gray and pale granulation.” These characteristics belong to the syndromes of “Xu (deficiency)” and “Yu (stasis).” The pathologic mechanisms underlying the refractory skin ulcers are that “long term disease results in a deficiency and stasis in both qi and blood, leading to a disorder of *ying* (nutrition) and *wei* (immunity) and skin dystrophy.” In addition, it is well-known that “long term illness contributes to the development of Yu (stasis) and Xu (deficiency) syndromes.” Indeed, the “Yu” syndrome in an ulcer is an external manifestation of the deficiency in the five “zang” organs and the stasis of qi and blood [6]. Hence, three pathologic factors of the “Re (heat),” “Xu (deficiency),” and “Yu (stasis)” sequentially or simultaneously contribute to the development and progression of CSU. The “Re (heat)” is the symptom of an ulcer, while the “Xu (deficiency)” and “Yu (stasis)” are the causative factors of CSU. Sometimes, they are reciprocal causation [7] because “Yu causes Xu and vice versa.” Apparently, the “Xu” and “Yu” are two key pathologic factors of the development of CSU. Therefore, clearance of “Fu” (removal of necrotic tissues) is just one way to retard the disease. In contrast, combination of supplement vacuity of “Xu (*qi blood and ying yang*)” with the removal of “Yu” is the fundamental basis for the treatment of CSU. From long term clinical practice and scientific researches, we believe that the “qu-yu (removal of stasis)” is crucial for the treatment of CSU, according to the therapeutic principle of “bu-xu-qu-yu (tonification of weakness and removal of stasis),” because the removal of the local stasis can eliminate the source of rotten tissue formation, promote the growth of new tissues, and limit the formation of scars [8]. Hence, we further emphasize the view that “removal of stasis is beneficial for the growth of new tissues with little scar” [9].

## 3. The Characteristics of Different Syndromes of Ulcers

In TCM, a syndrome is a complex disharmony pattern of signs and symptoms at a given stage of the development and progression of a disease, and it reflects the internal and external conditions of individuals as well as the pathogenesis, location, instincts, healthy energy-evil struggle, and degree of the disease. The syndromes of CSU are the comprehensive manifestation of systemic and local pathogenesis. Wang and Que [10] reported 338 patients with CSU at the lower limbs and divided the clinical syndromes into 3 types of qi and blood deficiencies, spleen deficiency with dampness encumbrance, and qi deficiency with blood stasis. Patients with qi and blood deficiencies are characterized by subsidence wound with pale or dim purple granulation, stiff dull skin around the ulcer, and fatigue to talk. Patients with spleen deficiency and dampness encumbrance usually have a swelling granulation on the wet wound, light yellow and thin pus, sallow complexion, and little defecation, while patients with qi deficiency and blood stasis commonly have pus immersion or pus covering tongue fur, lots of pus or rotten tissues with odor smell, heat pain or itch pain in center place, warm or a little hot skin, and dull-red skin around the wound. Our clinical researches have classified these ulcer patients into qi and blood deficiencies, spleen deficiency with dampness encumbrance, and qi deficiency with blood stasis.

## 4. Strategies for Systemic Treatment of Patients with CSU by the “Qing-Hua-Bu” Protocol

Modern medical science has divided the healing process of ulcers into the three phases of inflammation, tissue formation, and tissue remolding. During the healing process, there are two main aspects of the growth of new granulation in the wound and the migration of the epithelial tissue to cover the wound. In the history of TCM, there are different strategies for the treatment of different stages of CSU. Treatment of a CSU with TCM still follow the principle of “syndrome differentiation and treatment,” combining systemic regulation with local treatment and considering simultaneous interior and exterior treatments and the symptoms and causes together, according to the disease and its progressive stage. Methodologically, we should consider three major pathologic factors of “Re,” “Yu,” and “Xu” in the three stages of the pathogenic process of a CSU. On the basis of the common principle of “qu-fu-sheng-ji (removal of necrotic tissues to stimulate the growth of fresh skin)”, we should pay special attention to the supplement of “Xu” and the removal of “Yu” using the “qing-re (clearing heat), hua-yu (resolve stasis), and bu-xu (tonification)” methods to treat the syndromes at earlier, middle, and later stages of the disease, respectively. These three strategies can be applied independently, sequentially, or simultaneously.

### 4.1. Clearance of Heat and Promotion of Diuresis to Treat Dampness Invasion at the Early Stage of CSU

**Figure 1:**
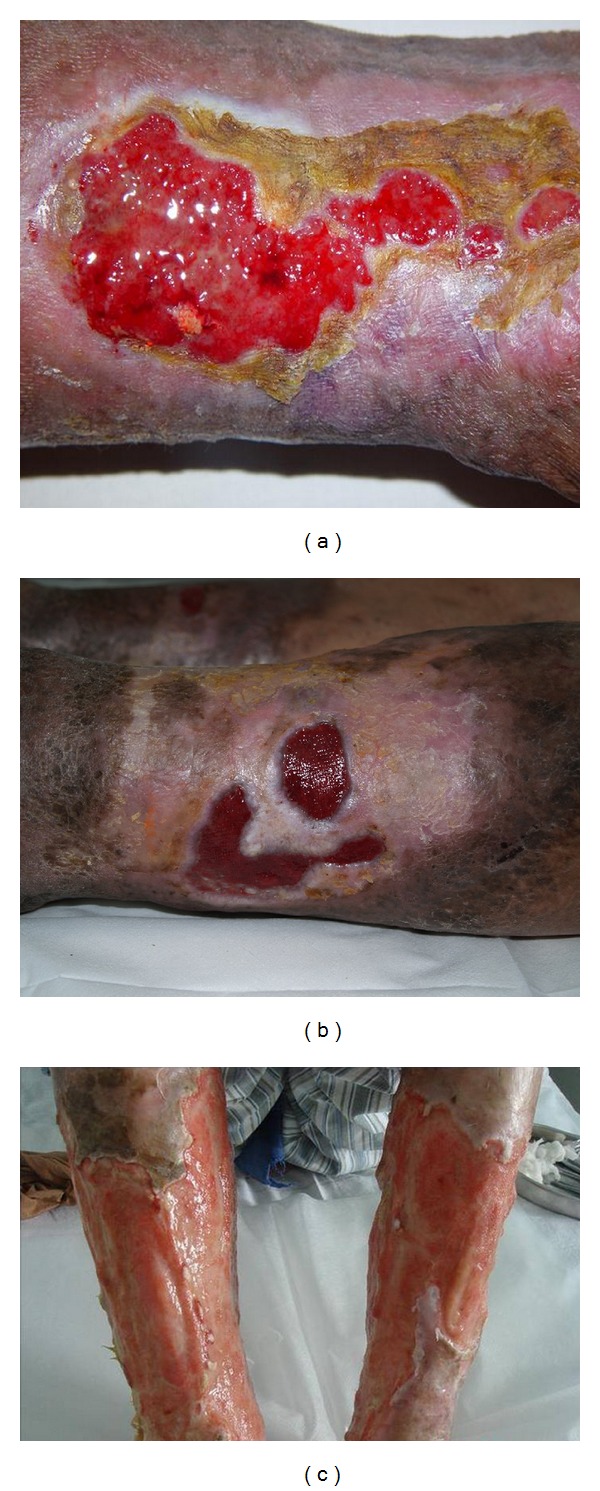
Typical cases with CSU. (a) A case with dampness-heat diffusing downward syndrome of CSU manifested with red granulation and yellow pus. (b) A case with qi-stagnancy and blood-stasis syndrome of CSU manifested with dark redness or dim purple skin around granular tissues. (c) A case with vital-Qi deficiency and blood stasis syndrome of CSU manifested with gray-white and swelling granulation.

At the early stage of CSU, foreign particles and bacteria in the wounded area will be cleared by the activated infiltrating neutrophils and be phagocytosed by macrophages. Patients with CSU at this stage usually show skin inflammation with local skin itching and pain, red swelling and diffusion, and progressively form an ulcer following the broken lesion and fluid secretion (Figure 1(a)). Inappropriate treatment may deteriorate the ulcer with fresh red meat accompanied by odorous pus, thick or thin ulcer edge, red or purple skin around the ulcer as well as heat burning and pain. Sometimes, patients with ulcers may have a red tongue with a thin or thick yellow coating and a slippery and string pulse. These symptoms result mainly from long-term standing or heavy loading that damage qi and blood circulation, leading to poor nutrition of the skin. This, together with the wound and the invasion of dampness heat and pathologic evil, will eventually cause the symptoms of ulcers. Hence, treatment of an ulcer at this stage should follow the principle of “clearing away heat and removing dampness, harmonizing *ying* for detumescence” using the modified Simiao Bolus plus Biye Shenshi decoction for drying dampness. We can use Jin Yin Hua (*Lonicera japonica* Thunb), Zi Hua Di Ding (*Viola chinensis*) for clearing away heat and detoxify; Cang Chu (*Atractylodes lancea*), Fu Ling (*Poria cocos*), Yi Yi Ren (*Semen coicis*), Han Fang Ji (*Stephania tetrandra* S. Moore) as the subordinate medicines to clear away dampness and detumescent, as well as to reduce the side effects of the principal medicines, including bitter cold and stomach-hurting. Dang Gui (*Angelica sinensis*), Chi Shao (*Platycodi Radix*), and Dan Pi (*Moutan Cortex*) are used as the assistant medicines to cool blood and resolve stagnation. Chuan Niu Xi (*Cyathula officinalis*), Huang Bo (*Phellodendri Chinensis Cortex*), and Sheng Gan Cao (*Glycyrrhizae Radix*) are used for leading heat downward flow and harmonizing all the medicines.

**Recommendations for Topical Treatments of an Ulcer**

If the wound is covered by pus or rotten tissues, we can spread Jiuyi, Ba'er, Qisan, or even Wuwu powder on the surface, depending on the thickness and tightness of the pus and rotten tissues. Subsequently, we cover the wound with a thin layer of Hongyou ointment (red greasy ointment) and fix the wound daily until the pus and decayed tissues in the ulcer disappear and the color of the granulation changes into red. If an ulcer is surrounded by red swelling and heat pain, we may use Ruyi golden ointment to compress around the skin, but not directly on the wound. Once the wound is red swelling with itching and produces a lot of fluid, we will use the Qing dai ointment. If an ulcer produces a lot of fluid, we will use the diluted skin health washing liquid. If there is a small wound with empty vesicle under the wound and a lot of fluid, we will use a drug thread to drain out the liquid.

### 4.2. Promoting Blood Circulation to Dissipate Blood Stasis for the Treatment of Patients with a CSU and a Qi-Stagnancy and Blood-Stasis Syndrome at the Middle Stage of the Wound-Healing Process

At the middle stage of the wound-healing process, inflammation reactivity in an ulcer is reduced and new stroma, which is often called granulation tissue, begins to grow until the wound space is filled. There is no decayed tissue and pus on the ulcer, but may have a crusting scar on the surface of the ulcer. The wound usually displays dark redness or dim purple around the granular granulation tissues (Figure 1(b)) with a hard base, but the wound does not minimize within days. The wound is surrounded by dim purple or dark gray skin with a dull and stiff feeling. The edge of the wound uplifts with a shape of the “mouth of water tank in China,” Meanwhile, the granulation tissues are usually hyperplastic, accompanied by numb and dull pain. Patients usually have stasis spots and ecchymosis on their dark red tongue with thick-white grease or yellow grease, and their pulse becomes stringy and slow or thin and obscure. Patients with a long-lasting ulcer at this stage usually have the evil qi in their bodies for a while, which stagnates the meridian and causes wound dystrophy. These alterations will result in growth stagnation, which in turn leads to the migration of the wound and a vicious circle of the healing process. Treatment should primarily consider to promote qi and blood circulation and to clear the stagnation by using the modified Taohong Siwu Decoction. In this decoction, Tao Ren (Peach seed), Hong Hua (*Carthamus tinctorius*), and Dang Gui (*Angelica sinensis*) are used as the monarch drugs to resolve stasis and circulate the blood. Chuan Xiang (*Aucklandiae Radix*), Ru Xiang (*Olibanum*), Mo Yao (*Comiphora Myrrha*), Chuang Xiong (*Rhizoma Chuanxiong*), and Di Long (*Pheretima*) are used as the ministerial drugs to circulate the meridian. Chi Shao (*Paeoniae Radix*), Dan Shen (*Salviae miltiorrhizae Radix*), and Sheng Di (*Rehmanniae Radix*) are used as the adjuvant drugs to cool the blood and for detoxifying. Gan Cao (*Glycyrrhizae Radix*) is used to harmonize all the medicines involved.

**Recommendations for Topical Treatments of an Ulcer at This Stage**

If we observe a wound covered with the crust or dry necrotic tissues, we would initially treat the wound with Vaseline greasy ointment to soften the crust within days, and then employ a minimal debridement method, just like a silkworm eating food to clear the crust. If we see an ulcer without pus and crust, we will spread Shengji Powder or Babao pill on the wound, followed by covering the wound with a gauze with Shengji Yuhong ointment, Shengji Bayu ointment, or red greasy ointment. If we observe a wound surrounded by the “mouth of the water tank in China” with a dim dark and stiff skin, we usually use a percussopunctator to stick the edge and the skin around the ulcer moderately and then use the strategies described above. If we detect hyperplastic granulation that is higher than the edge of the wound, which may block the epithelial growth, we would sift Pingnu pill or Wumei powder to the wound, use 10% NaCl hydropathic gauzes to compress the ulcer for several days, or directly use debridement method.

### 4.3. Supplements for Deficiency to Promote Granulation for the Treatment of Patients with a CSU and Vital-Qi Deficiency and Blood Stasis Syndrome at Later Stage of the Wound-Healing Process

At later stage of the wound-healing process, the wound will contract and be subjected to extracellular-matrix reorganization. Patients with a vital-qi deficiency and blood stasis syndrome usually display a subsided wound with pale or swelling granulation, but without obvious necrotic tissues. Their wounds can be surrounded by the “mouth of water tank in China” and purple, hard, and stiff skin, particularly in the afternoon. Their tongues are pale and fatty with teeth marks around the brim and ecchymosis as well as thin and white grease, and their pulses are string fine or heavy fine. These pathogenic characteristics usually come from long-term diseases. It is wellknown that “the long illness causes Xu, and the long illness causes Yu.” Actually, “Yu” can lead to “Xu” and “Xu” can enhance “Yu.” The qi deficiency inhibits the growth of fresh tissues and is associated with wound subsidence and gray-white and swelling granulation (Figure 1(c)). The qi deficiency also disturbs normal circulation and leads to wound swelling, particularly in the afternoon. Similarly, the heave “Yu” promotes the formation of the “mouth of water tank in China” around the wound with purple, hard, and stiff skin. The principle strategies for the treatment of CSU at this stage are to use medicines for the Yi-qi-huo-xue (supplementing qi and activating blood) and bu-xu-sheng-ji (tonification deficiency to promote tissue regeneration). A modified Buyang Huanwu Decoction should be applied for patients with a CSU at this stage. In this decoction, Huang Qi (*Astragalis Raw Radix*) and Tai Zi Shen (*Pseudostellariae Radix*) are used as the monarch drugs to strengthen healthy energy. Dang Gui (*Angelicae sinensis* Radix), Dan Shen (*Salviae miltiorrhizae* Radix), Tao Ren (*Semen Persicae*), Hong Hua (*Carthami Flos*), and Chuang Xiong (*Rhizoma Chuanxiong*) are used as the ministerial drugs to reduce stasis and to circulate the blood. Bai Zhu (*Rhizoma Atractylodis Macrocephalae*) and Fu Ling (*Poria Cocos*) are used as the adjuvant drugs to induce diuresis through strengthening the spleen. A leech is used to circulate the meridian. Chuan Niu Xi (*Cyathulae Radix*) is used to lead the downward drug flow. Gan Cao (*Glycyrrhizae Radix*) will harmonize all the medicines involved.

**Recommendations for Topical Treatments of an Ulcer at This Stage**

Due to the lack of necrotic tissues and pus on the wound at this stage, we can spread Sheng Ji powder and Babao pill on the wound and cover with a gauze with Hongyou ointment or Shengji Yuhong ointment. If there is a vesicle under the wound, we can compress the wound by a bound tied therapy. If the ulcer subsides, we can add cotton to ensure that the wound be exposed to drugs. If the wound displays dim-dry, hard, and stiff granulation, we can use ointments, such as Hongyou ointment, Shengji Yuhong ointment, and Shengji Baiyu ointment, as well as Tangchuang oil or Shirun Shao shang oil. If there are a lot of fluids on the ulcer, we can cover the wound with a gauze with Kangfuxin liquid or recombinant bovine fibroblast growth factor. In parallel with these treatments, physical treatments, such as microwave, far infrared energy, UV, and laser can be used as the assistant methods.

## 5. Treatment of the CSU-Related Primary and Associated Diseases

The refraction of CSU is mainly related to its primary disease and its related comorbid factors. Actually, CSU is not an independent disease, rather a syndrome of other diseases at a certain stage. Hence, inquiring a detailed history of the disease, careful physical examination, and completing auxiliary examinations, including etiologic examination and pathologic biopsy, to confirm the diagnosis and to detect the refractory factors prior to treatment will be helpful. These preliminary works will play critical roles in the treatment of patients with a CSU and preventing the formation of a hyperplasic scar. In addition, these procedures will help in the diagnosis of malignant diseases or specific infection and in making appropriate decisions for the treatment of a CSU.

It is important to change position of patients with a pressure ulcer at least every 1-2 hours to relieve the pressure using pillows, sheepskin, foam padding, and others, because the long-term pressure against the skin reduces blood supply to the area, accompanied by active treatment of patients who have malnutrition, imbalance of electrolytes, and other basic diseases simultaneously. For a venous ulcer, we usually suggest that the patients reduce the standing time and avoid walking for a long time. Actually, simple elevation of their legs above heart level for 30 minutes three to four times per day can also reduce edema and improve blood flow in the veins, which can accelerate the healing process. We also suggest that the patients wear elastic socks and bandages in combination with some drugs that improve venous blood circulation, such as Aescuven forte, melilotus extract tablet, and Diosmin. Moreover, we pay special attention to treatment of deep vein thrombosis and lower limb embolism.

If patients have arterial ulcers, we first differentiate ulcers that stem from diabetic angiopathies and thromboangiitis obliterans from arteriosclerosis obliterans. Subsequently, we treat the patients with a vasodilator and platelet aggregation inhibitors such as Alprostadil, cilostazol, or low molecular dextran as well as TCM with a function of promoting blood circulation. If necessary, we will provide an operation and interventional therapy for those patients. If patients have an ulcer secondary to allergic cutaneous vasculitis or pyoderma gangrenosum, we will treat the primary disease actively. If bacterial test is positive for a specimen from the ulcer, we will distinguish the colonization of a bacterium from infective bacteria in the wound, because the presence of bacteria in a wound can be a sample contamination, bacterial colonization, critical colonization, or infection. Actually, all chronic wounds are contaminated, and they contain at least nonreplicating microorganisms within or on the surface of the wound bed. Microorganisms live in a wound, such as bacterial colonization, and can be harmless because host defenses can clear them out. Colonization is defined as replicating microorganisms that adhere to the wound surface, but do not cause damage to the host (Wound and Skin Care Center; http://www.vnaa.org/vnaa/g/?h=html/wound_center_July). Hence, it is not necessary to provide special treatment for them. Infection characterized by the presence of replicating microorganisms within a wound will result in a subsequent host response. If bacterial infection causes high fever and accounts of white blood cells as well as erythema, warmth, swelling, pain, odor, and purulent drainage around the wound, these suggest that the infected bacteria may have entered into the body, leading to a systemic infection. Accordingly, we usually treat these patients with antibiotics or other medicines, according to the antibiotic sensitivity of the microorganism, particularly for those with fungus, *Mycobacterium tuberculosis*, or atypical *Mycobacterium* infection. Patients with diabetes for a long period usually develop vascular complications and neuropathy so that their ulcers are easily secondary to bacterial infection. Therefore, therapeutic strategies should include hyperglycemic correction, peripheral vascular vasodilation, neuron nutrition, and controlling infection. In addition, we should recognize that long-term unsealed skin ulcers may undergo malignant transformation. Similarly, an ulcer can be a symptom of some skin malignant tumors. Hence, it is critical to recognize the possibility of malignant tumor with marginal swelling, cauliflower shape, and hardness around the ulcer tissue, particularly for a long-term unsealed ulcer. We recommend multipoint and multitime pathological biopsies for a highly suspicious ulcer to affirm the diagnosis, and then perform an operation or other radio and chemotherapies for those patients.

Numerous data from many animal and clinical studies implicate the efficacy of TCM in the treatment of a CSU [11–16]. However, there is a little evidence to demonstrate the efficacy of TCM in the treatment of a diabetes-related CSU in the clinic due to the lack of high-quality clinical researches. Therefore, further high-quality clinical investigations are warranted to verify the beneficial activity of TCM in the treatment of a CSU [17].

## Abbreviations

TCM: Traditional Chinese medicine
CSU: Chronic skin ulcers.
